# Consumption of diets high in prebiotic fiber or protein during growth influences the response to a high fat and sucrose diet in adulthood in rats

**DOI:** 10.1186/1743-7075-7-77

**Published:** 2010-09-29

**Authors:** Alannah D Maurer, Lindsay K Eller, Megan C Hallam, Kim Taylor, Raylene A Reimer

**Affiliations:** 1 Department of Biochemistry and Molecular Biology, University of Calgary, 3280 Hospital Drive NW, Calgary, T2N 4Z6, Canada; 2 Faculty of Kinesiology, University of Calgary, 2500 University Drive NW, Calgary, T2N 1N4, Canada

## Abstract

**Background:**

Early dietary exposure can influence susceptibility to obesity and type 2 diabetes later in life. We examined the lasting effects of a high protein or high prebiotic fiber weaning diet when followed by a high energy diet in adulthood.

**Methods:**

At birth, litters of Wistar rats were culled to 10 pups. At 21 d pups were weaned onto control (C), high prebiotic fiber (HF) or high protein (HP) diet. Rats consumed the experimental diets until 14 wk when they were switched to a high fat/sucrose (HFHS) diet for 6 wk. Body composition and energy intake were measured and an oral glucose tolerance test (OGTT) performed. Blood was analyzed for satiety hormones and tissues collected for real-time PCR.

**Results:**

Weight gain was attenuated in male rats fed HF from 12 wk until study completion. In females there were early reductions in body weight that moderated until the final two wk of HFHS diet wherein HF females weighed less than HP. Final body weight was significantly higher following the high fat challenge in male and female rats that consumed HP diet from weaning compared to HF. Lean mass was higher and fat mass lower with HF compared to HP and compared to C in males. Energy intake was highest in HP rats, particularly at the start of HFHS feeding. Plasma glucose was higher in HP rats compared to HF during an OGTT. Plasma amylin was higher in HF females compared to C and glucagon-like peptide-1 (GLP-1) was higher in HF rats during the OGTT. Leptin was higher in HP rats during the OGTT. HF upregulated GLUT 5 mRNA expression in the intestine and downregulated hepatic hydroxymethylglutaryl coenzyme A reductase. Male rats fed HP had higher hepatic triglyceride content than C or HF.

**Conclusion:**

These data suggest that while a long-term diet high in protein predisposes to an obese phenotype when rats are given a high energy diet in adulthood, consumption of a high fiber diet during growth may provide some protection.

## Background

The prevalence of obesity has increased dramatically over the past two decades and is in turn contributing to the increased rates of comorbidities, particularly type 2 diabetes [1]. In children, the prevalence of obesity doubled and tripled in many countries between the 1970's and the 1990's [2]. Obesity in children has been linked to alterations in glucose metabolism which can lead to type 2 diabetes. Obesity is also an early risk factor for much of adult morbidity and mortality [3]. The best approach to preventing and/or treating obesity remains elusive.

A diet high in fiber is associated with various health benefits including reduction in colorectal cancer risk [4]. Fiber intake is also inversely associated with body mass index (BMI) and risk of type 2 diabetes [5,6]. Dietary recommendations for both the management and prevention of type 2 diabetes include high dietary fiber intake [7]. Various fiber sources have demonstrated benefits, including diets high in rye fiber blunting plasma glucose and insulin peaks [8] and psyllium fiber attenuating weight gain in mice fed a high-fat diet [9]. We have previously shown that a highly fermentable fiber diet in rats increases the secretion of glucagon-like peptide (GLP-1), a potent insulin secretagogue that also slows gastric emptying, inhibits glucagon secretion, enhances β-cell proliferation and regulates food intake [10,11].

High protein diets have become increasingly popular for weight loss [12]. The purported benefits of a high protein diet include increased satiety, increased thermogenesis and the maintenance of fat-free mass during weight loss [13]. Weight loss diets high in protein have also been associated with reductions in abdominal fat and low density lipoprotein cholesterol [14], reduction in serum triacylglycerol concentrations [15] and an improvement of cardiovascular risk profile [16]. Some evidence would suggest, however, that the satiating effect of protein is reduced with habitual protein intake [17].

It is now increasingly clear that response to diet in adulthood is programmed in part by the nutritional influences experienced during growth and development. A given genotype can give rise to distinct phenotypes depending on environmental conditions during development [18]. Exposure to specific nutritional environments during critical periods of development have long-term repercussions for health in adulthood, including increased risk of obesity and type 2 diabetes [19]. This 'developmental plasticity' is important as it allows a fetus to make adaptations that will favor its best chance of survival in the environment it expects to encounter after birth [20]. We now know that this plasticity continues well past birth [21] and nutrient intake in both pre- and postnatal periods can shape development. In addition, there is evidence suggesting that a "mismatch" between early environment (dietary or otherwise) and an organism's later environment can increase disease risk [22].

Independently, diets high in fiber or protein can contribute to various positive metabolic outcomes in adulthood depending on the condition they target. Whether or not long-term diets high in fiber or protein introduced at weaning provide protection against a high energy diet (a mismatched environment) in adulthood is not known. We have previously shown that high protein and high fiber diets introduced at weaning have differential effects on the expression of genes involved in glucose and lipid metabolism and inflammation in rats at 21 to 35 days of age [23]. Plasma GLP-1 was higher and leptin lower in young rats consuming a high fiber compared to a control or high protein diet [23]. Whether or not the pattern of gene expression and satiety hormone production established in young rats persists into adulthood when a high fat/sucrose diet challenge is given is not known. Therefore, our objective was to examine body weight, fat mass and the expression of satiety hormones and genes related to glucose and lipid metabolism in rats consuming a control, high fiber or high protein diet until adulthood and then undergoing a metabolic challenge with a high energy diet.

## Methods

### Animals and diets

Female Wistar rats were obtained from Charles River (Montreal, PQ, Canada) and housed in a temperature and humidity controlled room with a 12-h light/dark cycle. Following acclimatization, females were mated with male Wistar rats in wire-bottom cages to aid in identification of a copulation plug. Females were then isolated and given ad libitum access to control diet. All litters were culled to 10 pups (5 males and 5 females where possible) one day following birth to promote consistency of nourishment between litters during the suckling period. At weaning (21 d), males and females were separated and 6 litters were placed on each of 3 experimental diets: control (C), high prebiotic fiber (HF, 22% wt/wt) and high protein (HP, 40% wt/wt). Composition of the diets is provided in Table 1. The HF diet used a combination of the prebiotic fibers, inulin and oligofructose, at a ratio of 1:1 (by weight). The prebiotic fiber blend was mixed in our laboratory but resembles commercially available Synergy 1 (Orafti BENEO, Tienen, Belgium). Rats consumed these diets until 14 wk of age (equivalent to young adulthood) when all rats were given a high fat/high sucrose (HFHS) diet for 6 weeks. The HFHS diet provided 40% of energy from fat and 45% from sucrose and was composed of (g/100 g): cornstarch (5); casein (14), sucrose (51), soybean oil (10), lard (10), Alphacel (5), AIN-93 M mineral mix (3.5), AIN-93 vitamin mix (1), L-cystine (0.3), and choline bitartrate (0.25). Adjusting the cornstarch component of the experimental weaning diets has been used previously [23,24] and results in minimal change to other essential nutrients. Food and water were provided ad libitum throughout the experiment. Principles of laboratory animal care were followed. The protocol was approved by the University of Calgary Animal Welfare Committee and conformed to the Guide for the Care and Use of Laboratory Animals.

**Table 1:** Experimental diet composition

Composition (g/kg)	Control*	High Protein	High Fiber
Cornstarch	465.7	205.7	325.5
Casein	140	400	120.3
Dextrinized Cornstarch	155	150	133.3
Sucrose	100	100	86
Soybean Oil	40	40	34.4
Alphacel	50	50	43
AIN-93-MX	35	35	30.1
AIN-93-VX	10	10	8.6
L-Cystine	1.8	1.8	1.6
Choline bitartrate	2.5	2.5	2.2
Inulin/Oligofructose^Ψ^	0	0	215

*Based on AIN-93 M purified diet to meet all nutritional requirements. The digestible energy of the control and high protein diets is 3.6 kcal/g and the high fiber diet slightly lower at 3.2 kcal/g due to the reduced energy content of the inulin and oligofructose.

^Ψ^Inulin supplied as Orafti Raftiline HP and oligofructose as Orafti Raftilose P95 in a 1:1 blend by weight (Quadra Chemicals Ltd., Burlington, ON, Canada). The energy value of the fiber blend (1.5 kcal/g) was used to substitute for an equicaloric amount of cornstarch in the high fiber diet.

### Food Intake and Body Composition

Food intake was measured daily for a week every two weeks throughout the study by weighing each food cup to the nearest 0.1 g and then subtracting this weight from the previously measured weight. Body weight was measured once a week throughout the study. At the end of the six week HFHS feeding period, final body weight was measured and body composition determined under light anaesthesia using dual energy x-ray absorptiometry (DEXA) with software for small animal analysis (Hologic QDR 4500, Hologic, Inc., Bedford, MA).

### Oral glucose tolerance tests for glucose and satiety hormone levels

One week prior to the end of the study, rats were feed-deprived overnight and a fasted blood sample taken vial tail nick. An oral glucose gavage (2 g/kg) was administered and additional blood samples taken at 15, 30, 60, 90, and 120 min. Blood glucose was measured immediately using a One Touch Blood Glucose Meter (LifeScan, Inc, Milpitas, CA). At the end of the study, a second OGTT was performed for satiety hormone analysis. After an overnight fast, rats were anaesthetized with isoflurane and a fasting cardiac blood sample taken. Rats were allowed to wake and given an oral load of 2 g/kg glucose. Subsequent blood samples were collected into chilled vacutainers (BD Biosciences, Mississauga, Ontario, Canada) with the addition of EDTA (1 mg/mL), aprotinin (5 × 105 KIU/L) and the DPP-IV inhibitor, diprotin A (34 μg/ml; Calbiochem, La Jolla, CA) at 15, 30, and 60 min post-gavage. Plasma was stored at -80°C until later analysis. After the final blood sample, the rats were over-anaesthetized and the cervical spine dislocated. The small intestine was excised, flushed, measured and weighed, then divided into three segments designated duodenum, jejunum and ileum. A distal piece of each section was immersed in liquid nitrogen and stored at -80°C for later mRNA analysis. The colon, stomach, liver and cecum were also excised, measured, weighed, and stored at -80°C for mRNA expression analysis.

### Plasma analysis

A multiplex hormone assay kit and Luminex instrument were used to measure plasma insulin, leptin, total amylin and glucagon concentrations (Rat Endocrine LincoPlex Kit, Millipore, St. Charles, MO). The sensitivity of the multiplex kit was 55.6 pM for insulin and 6.2 pM for all other analytes. Concentrations of active GLP-1 were measured using a GLP-1 (Active) ELISA kit (LINCO Research, Millipore, St. Charles, MO). The lowest level of GLP-1 that can be detected by this assay is 2 pM.

### RNA extraction and Real-Time RT-PCR

Total RNA was extracted from the stomach, small intestine, colon and liver using TRIzol reagent (Invitrogen, Carlsbad, USA). Reverse transcription was performed with an input of 1 μg of total RNA using the 1st strand cDNA synthesis kit for RT-PCR (Invitrogen, Carlsbad, CA USA) with oligo d(T)15 as a primer. The resultant cDNA was amplified using primers synthesized by the University of Calgary Core DNA Services (Calgary, AB, Canada) and analyzed by real time PCR. Primer sequences were according to our previous work [23]. The PCR reaction was heated for 1 min 30 s then 40 cycles at 95°C for 30 s, 60°C for 30 s and 72°C for 20 s in an iCycler instrument (BIO-RAD, Hercules, USA). A melt curve showed the melting point of the PCR product of interest. Actin was verified as a suitable housekeeping gene for the tissues of interest and actin primers included as an internal control in the reactions. The 2^-ΔCT ^method [ΔCT = CT (gene of interest) - CT (reference gene)] was utilized for the data analysis where threshold cycle (CT) indicates the fractional cycle number at which the amount of amplified target reaches a fixed threshold [25]. The ΔCT is the difference in threshold cycles for the gene of interest and actin.

### Hepatic triglyceride and cholesterol content

Triglyceride and cholesterol contents in samples of liver tissue were measured according to our previously published protocol [26]. Values are expressed as mg of triglyceride or cholesterol per mg of protein in the liver samples.

### Statistics

All data are presented as mean ± SE. A multivariate ANOVA was used to evaluate differences between groups with post hoc tests using Bonferroni correction. Diet and sex were included as fixed factors for physical characteristics, gene expression data, triglyceride and cholesterol content. Changes in glucose and hormone levels during the OGTT and longitudinal body weight and energy intake data were analyzed with repeated measures ANOVA. Throughout the analysis, when no sex differences were found data was combined for further analysis. Differences were considered significant at p ≤ 0.05. Statistical analyses were performed using SPSS v 16.0 software (SPSS Inc., Chicago, IL).

## Results

### Body composition, energy intake and organ weights

**Figure 1:**
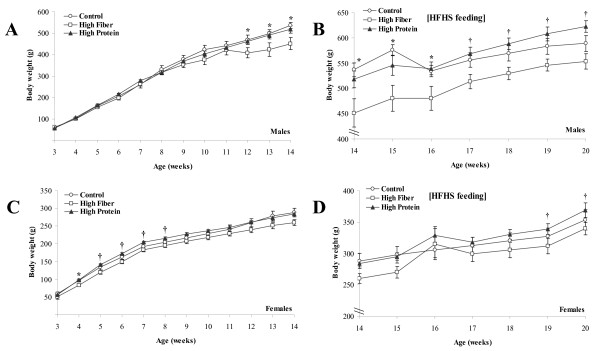
**Body weight of male and female rats that consumed a C, HF or HP diet from weaning until 14 wk of age and were then switched to a HFHS diet for 6 wk**. Results are presented as mean ± SE, n = 10 per group. Panel A provides the longitudinal measures of body weight in male rats that consumed C, HF or HP diet from weaning (3 wk) until young adulthood (14 wk). Panel B provides body weight for male rats when switched to HFHS diet for 6 wk until 20 wk of age. Panel C provides longitudinal body weight in female rats that consumed C, HF or HP diet from weaning (3 wk) until young adulthood (14 wk). Panel D provides body weight for female rats when switched to HFHS diet for 6 wk until 20 wk of age. In all Panels, the * represents a difference (p < 0.05) between HF versus C and HP at the indicated time point. The † represents a difference (p < 0.05) between HF and HP.

**Figure 2:**
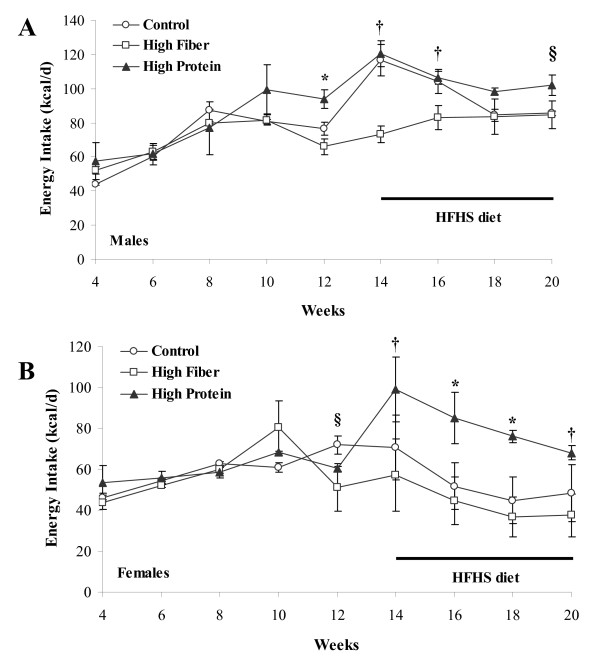
**Energy intake of male and female rats that consumed a C, HF or HP diet from weaning until 14 wk of age and were then switched to a HFHS diet for 6 wk**. Results are presented as mean ± SE, n = 5 per group. Panel A provides the energy intake in male rats measured daily for a week every two weeks throughout the study. Rats were switched from their weaning diet (C, HF or HP) to HFHS at 14 wk of age and consumed it until study completion at 20 wk of age. Panel B provides the energy intake in female rats measured for daily for a week every two weeks throughout the study. In Panel A, the * represents a difference (p < 0.05) between HP versus HF and C. The † represents a difference (p < 0.05) between HF versus HP and C. The § represents a difference (p < 0.05) between HP versus HF. In Panel B, the * represents a difference (p < 0.05) between HP versus HF and C. The † represents a difference (p < 0.05) between HF versus HP. The § represents a difference (p < 0.05) between HF versus C.

Body weight was similar across all groups of male rats until 12 wk of age when the magnitude of weight gain slowed significantly in the HF fed rats compared to HP and C (p < 0.05, Figure 1A). When the rats were switched to a HFHS diet (Figure 1B), HF rats had significantly lower body weight than C and HP at 15 and 16 wk of age (p < 0.05). From 17 wk to the end of the study, HP were significantly heavier than HF rats (p < 0.05). In females, the HF diet was associated with a lower body weight at early time points than in the males (Figure 1C). At 4 wk of age, HF rats had lower body weight than C and HP rats while in weeks 5 through 8, HF was lower than HP (p < 0.05). The magnitude of difference in weight gain was smaller in females compared to males when switched to the HFHS diet (Figure 1D). At 19 and 20 wk of age, the HP female rats were significantly heavier than HF rats (p < 0.05). Final body weight in male and female rats raised on the HP was higher than the HF group (Table 2). Body fat was higher in HP male (p = 0.001) and female (p = 0.005) rats compared to HF and higher than C (p = 0.001) in males. Percent lean mass was significantly lower in HP compared to C and HF in males (p = 0.001). In females, HP was lower than HF (p = 0.003) but not C. Weight gain in male rats while they consumed the C, HF, or HP diet was significantly lower in HF versus C and HP rats (419 ± 10 g; 474 ± 10 g; 496 ± 13 g respectively in HF, C, and HP; p = 0.001). When switched to the HFHS diet for 6 wk, weight gain remained lower in HF versus HP rats (45 ± 6 g; 57 ± 6 g; 64 ± 4 g respectively in HF, C, and HP; p = 0.07). In females, the lower weight gain that occurred during wk 3 to wk 14 and during HFHS feeding was not significantly different from C and HP. Energy intake was higher in HP males compared to HF and C at 12 wk of age (p = 0.02, Figure 2A). HF rats had lower energy intake at 14 and 16 wk compared to C and HP and lower than HP at wk 20 (p < 0.05). In females, energy intake was lower in HF rats at wk 12 compared to C (p = 0.05, Figure 2B) and lower than HP rats at 14 and 20 wk (p < 0.05). HP rats had high energy intake at 16 and 18 wk compared to C and HF rats (p = 0.04).

**Table 2:** Body composition in rats switched from control, high fiber or high protein weaning diets to a high fat, sucrose diet in adulthood.

	Control		High Fiber		High Protein	
	Male	Female	Male	Female	Male	Female
Body weight (g)	589.0 ± 9.6^ab^	353.5 ± 6.1^ab^	553.1 ± 8.3^a^	339.7 ± 6.9^a^	622.4 ± 9.9^b^	369.4 ± 8.6^b^
Body fat (g)	144.1 ± 5.8^a^	114.6 ± 3.5^ab^	133.6 ± 5.4^a^	93.1 ± 5.3^a^	224.7 ± 22.3^b^	139.3 ± 19.2^b^
Lean mass (%)	75.5 ± 1.0^a^	67.6 ± 1.0^ab^	76.0 ± 0.8^a^	72.6 ± 1.5^a^	64.0 ± 2.2^b^	62.3 ± 2.8^b^

Values are mean ± SE with n = 9-10 per group. Lean mass represents lean tissue plus bone mineral content. Two-way ANOVA revealed a significant sex (p < 0.005) and diet (p < 0.001) effect for all three parameters. Treatments with different letters are significantly different between diets within males or females respectively (p < 0.05).

Organ weights, expressed as a proportion of individual total body weight, were calculated and are presented in Table 3. For both males (p = 0.003) and females (p = 0.001), adjusted small intestine length was greater with HF than HP and C. Small intestine weight was greater in HF (p < 0.001) and HP (p = 0.05) compared to C in males. In females, HF was greater than C (p = 0.01) and HP (p = 0.04). Colon length was greater with HF compared to C (p < 0.001) in females only. The HF diet resulted in significantly higher colon weight than the C (p < 0.01) and HP groups (p < 0.01) in both males and females. Empty cecum weight was significantly higher in rats consuming a HF diet compared to C and HP in males and females (p < 0.001).

**Table 3:** Body weight adjusted intestinal characteristics of rats switched from control, high fiber or high protein weaning diets to a high fat, sucrose diet in adulthood

Parameter	Control		High Fiber		High Protein	
	Male	Female	Male	Female	Male	Female
Liver Weight (mg/g)	21.6 ± 0.3	21.2 ± 0.7	21.3 ± 0.7	23.3 ± 0.7	23.2 ± 0.8	20.4 ± 0.7
Stomach Weight (mg/g)	4.9 ± 0.5	4.8 ± 0.3	4.2 ± 0.1	5.4 ± 0.3	4.2 ± 0.2	5.2 ± 0.3
Small Intestine Length (mm/g)	2.09 ± 0.04^a^	3.27 ± 0.01^a^	2.31 ± 0.03^b^	3.63 ± 0.01^b^	2.01 ± 0.04^a^	3.19 ± 0.01^a^
Small Intestine Weight (mg/g)	11.2 ± 0.2^a^	16.2 ± 0.3^a^	12.9 ± 0.3^b^	16.5 ± 0.3^b^	12.2 ± 0.2^b^	15.7 ± 0.3^a^
Colon Length (mm/g)	0.32 ± 0.01	0.38 ± 0.03^a^	0.36 ± 0.01	0.60 ± 0.03^b^	0.32 ± 0.01	0.52 ± 0.03^ab^
Colon Weight (mg/g)	2.5 ± 0.1^a^	3.4 ± 0.2^a^	3.3 ± 0.2^b^	4.2 ± 0.2^b^	2.6 ± 0.1^a^	3.4 ± 0.2^a^
Empty Cecum Weight (mg/g)	1.2 ± 0.1^a^	2.0 ± 0.2^a^	2.7 ± 0.2^b^	3.3 ± 0.2^b^	1.3 ± 0.1^a^	1.7 ± 0.2^a^

Values are mean ± SE with control (n = 9 M; n = 9 F); high fiber (n = 10 M; n = 10 F); and high protein (n = 10 M; n = 10 F). There was a significant diet effect (p < 0.01) for small intestine length and weight, colon length and weight, and cecum weight as determined with two-factor ANOVA. There was a significant sex effect (p < 0.01) for small intestine length and weight, colon length and weight, stomach weight, and cecum weight as determined by two-way ANOVA. There was a significant diet by sex effect for colon length (p = 0.008). Values with different letters are significantly different (p < 0.05) within males or females.

### Plasma hormones and blood glucose

**Figure 3:**
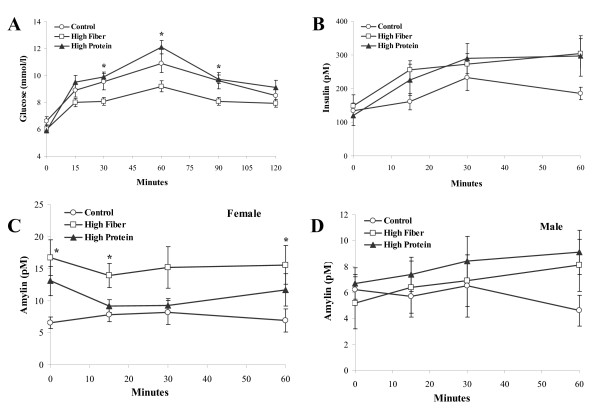
**Blood glucose and plasma insulin and amylin in rats during an oral glucose tolerance test following a high fat/sucrose diet challenge**. Results are presented as mean ± SE, n = 8-9 per group. Panel A provides the serial values of blood glucose during the OGTT. No sex effect was detected for glucose or insulin, therefore male and female data were combined. The * in Panel A represents a difference (p < 0.05) between HF versus HP and C. Panel B provides the serial values of plasma insulin. Panel C provides the serial values of amylin in female rats. In Panel C, the * represents a difference (p < 0.05) between HF and C at the indicated time points. Panel D provides the serial values of amylin in male rats.

**Figure 4:**
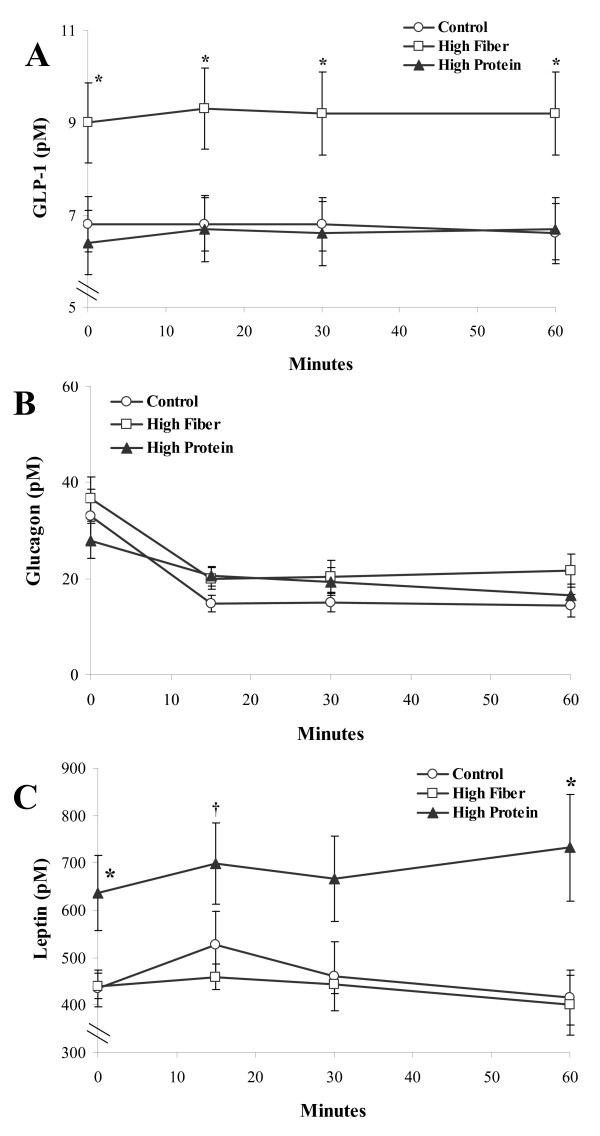
**Plasma GLP-1, glucagon and leptin in rats during an oral glucose tolerance test following a high fat/sucrose diet challenge**. Results are presented as mean ± SE, n = 8-9 per group. No sex effect was detected for GLP-1, glucagon and leptin, therefore male and female data were combined. Panel A provides the serial values of plasma GLP-1 during the OGTT. In Panel A, the * represents a difference (p < 0.05) between HF and HP at the indicated time points. Panel B provides the serial values of plasma glucagon. Panel C provides the serial values of leptin. In Panel C, the * represents a difference (p < 0.05) between HP versus HF and C. The † represents a difference (p < 0.05) between HP and HF at 15 minutes.

There was a significant diet effect for glucose wherein concentrations were higher for HP rats compared to HF at time 30, 60, and 90 min (p < 0.05; Figure 3A). There were no significant differences in insulin between the groups (Figure 3B). A significant sex effect was found for amylin therefore male and female data were analysed separately. There was a significant diet effect for amylin in female rats (p = 0.014; Figure 3C) but not male rats (Figure 3D). HF was greater than C in females at time 0 (p = 0.01), 15 (p = 0.02), and 60 minutes (p = 0.05). GLP-1 was significantly higher in HF rats than HP throughout the entire OGTT (p < 0.05; Figure 4A). Glucagon release was suppressed following the oral glucose load but did not differ between groups (Figure 4B). There were significant diet effects for leptin wherein HP was greater than HF and C at time 0 and time 60 (p < 0.05), and greater than HF at time 15 (p < 0.05; Figure 4C).

### Intestinal and hepatic gene expression

**Figure 5:**
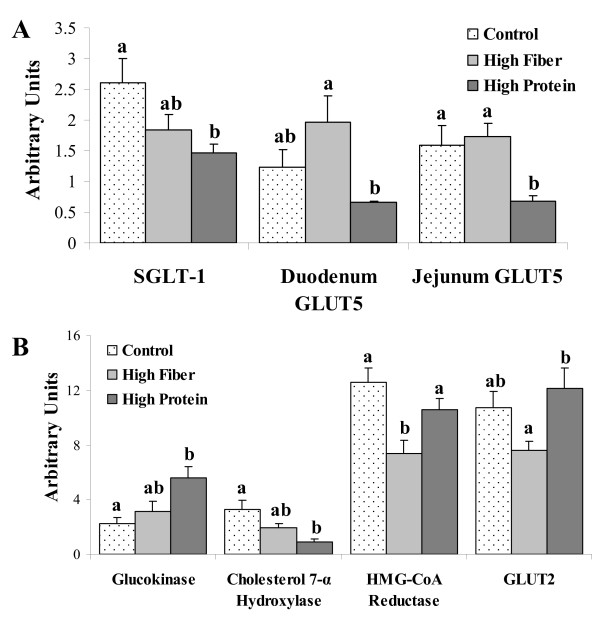
**Gene expression in the intestine (A) and liver (B) of rats challenged with a high fat/sucrose diet following HP, HF or C diets**. Results are presented as mean ± SE, n = 9-10 per group. No sex effect was detected, therefore male and female data were combined. Diet treatments with different letters within a gene represent a significant difference (p < 0.05). Sodium-dependent glucose transporter (SGLT-1), glucose transporter (GLUT); hydroxymethylglutaryl coenzyme A reductase (HMG-CoA reductase.

In the duodenum, HP was associated with lower SGLT-1 mRNA expression compared to C (p < 0.05; Figure 5A). HF was associated with an increase in the glucose transporter, GLUT5 mRNA compared to HP (p = 0.012). In the jejunum, both C (p = 0.026) and HF (p = 0.009) were associated with greater GLUT5 mRNA expression than HP. There were no differences in intestinal proglucagon mRNA expression. In the liver, HF was associated with a significant reduction in hepatic hydroxymethylglutaryl coenzyme A (HMG-CoA) reductase mRNA compared to C (p = 0.001) and HP (p = 0.05) (Figure 5B). The expression of hepatic cholesterol 7-α hydroxylase (CYP) mRNA was lower (p = 0.006) in rats fed HP compared to C. The HP diet was associated with higher expression of GLUT2 mRNA compared to HF (p = 0.03), and higher glucokinase mRNA compared to C (p = 0.03). There were, however, no significant diet differences in acetyl CoA carboxylase (ACC), fatty acid synthase (FAS), sterol regulatory element binding protein (SREBP)-1c, SREBP-2, lecithin cholesterol acyl transferase (LCAT) or leptin mRNA in the liver.

### Hepatic triglyceride and cholesterol content

**Figure 6:**
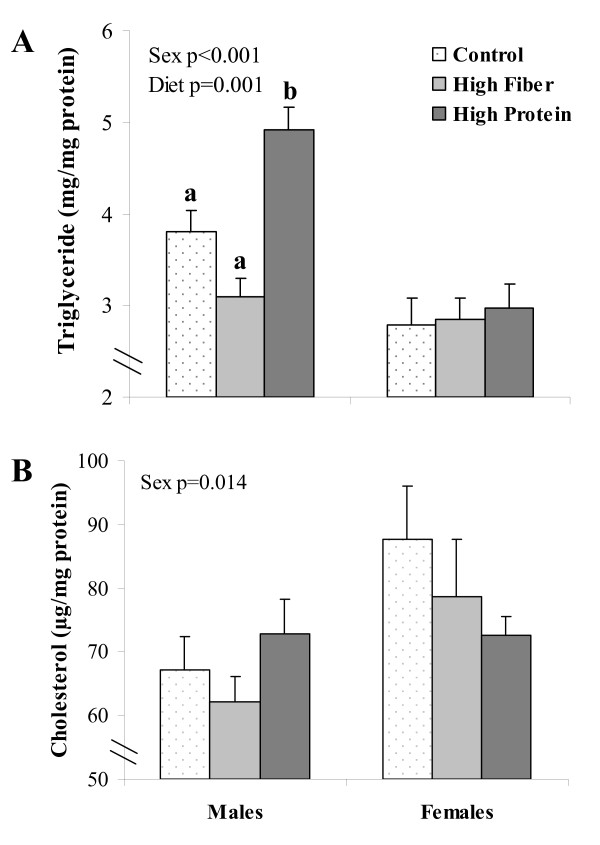
**Hepatic triglyceride and cholesterol content in rats challenged with a high fat/sucrose diet following HP, HF or C diets**. Results are presented as mean ± SE, n = 9-10 per group. Significant sex and diet effects are indicated in the graphs. Diet treatments with different letters within a gene represent a significant difference (p < 0.05).

There were significant sex (p < 0.001) and diet (p = 0.001) effects for hepatic triglyceride content (Figure 6). In male rats, HP had higher triglyceride content than C (p = 0.021) and HF (p = 0.001). There was a significant sex effect (p = 0.014) for cholesterol content but no diet effect.

## Discussion

As obesity rates escalate, it has become imperative to identify factors that contribute to the development of obesity and its comorbidities. The risk of developing the metabolic syndrome, a combination of risk factors predisposing individuals to cardiovascular and type 2 diabetes, has been shown to be particularly sensitive to nutritional influences early in life [27]. Most animal studies have used two major models of metabolic programming, that of maternal undernutrition to probe prenatal influences [28], and litter size manipulation to examine postnatal under- and overnutrition during the suckling period [29]. Much less work has examined the effects of maternal overnutrition and even less work has probed the effect of increased intake of select nutrients in the early postnatal period. Therefore, we examined the consequences of consuming a high prebiotic fiber or high protein diet throughout growth followed by a high energy diet challenge in adulthood.

The present study demonstrates that a long-term weaning diet high in fiber or protein results in significant differences in body weight and fat mass, the secretion of select satiety hormones, and the expression of genes involved in glucose and lipid metabolism in response to a high energy diet in adulthood. Our major findings include: 1) an increase in body weight and fat mass in response to high energy diets after a weaning diet high in protein compared to fiber; 2) an increase in energy intake in rats fed a HP diet, especially when switched to a HFHS diet; 3) a decrease in glucose and increase in GLP-1 in rats fed HF; and 4) greater accumulation of triglyceride in the liver of HP-fed rats. Take together, this data suggest that the response to a high energy diet challenge in adulthood results in greater metabolic dysfunction (ie. increased fat mass, decreased glucose tolerance, reduced GLP-1 secretion, greater hepatic triglyceride content) when a HP diet is consumed from weaning into early adulthood compared to a HF diet.

The increase in the relative mass of the small intestine, colon and cecum in the HF group is consistent with our previous work with fiber-enriched diets [10,11,24]. Addition of readily fermentable fiber to a diet is known to cause a significant proliferative effect in the colon and distal small intestine [30]. The prebiotic fibers, inulin and oligofructose, are highly water soluble and non-viscous and stimulate lactic-acid bacteria growth in the gut [31]. Inulin, chiefly derived from chicory, and its hydrolysis product, oligofructose, are fructans that have β-(2-1) linkages that differ in a high (inulin) and low (oligofructose) number of fructose molecules [32]. Reductions in body weight and fat mass [33], enhanced satiety [34], improved glucose control in hyperglycaemic subjects [35], increased GLP-1 secretion [36] and improved blood lipid profiles have all been demonstrated in humans consuming oligofructose [37].

GLP-1 is released from L cells in the intestine in response to food ingestion and has been shown to slow gastric emptying and decrease food intake and body weight [38]. We have previously shown that a diet high in fiber increases plasma GLP-1 concentrations [10,11,24]. There is some controversy surrounding the role of anorexigenic hormones, including GLP-1, in enhancing satiety with HP diets [39]. This study demonstrates that HF caused a significant sustained release of GLP-1 which was not observed in the HP or C rats. This increase is in agreement with work by others feeding oligofructose [40-42]. Similarly, amylin, a pancreatic β-cell hormone that inhibits food intake [43] was also increased by the HF diet in females. No significant differences between groups were seen in males which is likely due to the large variability observed in the males. Our observations of increased amylin in the HF-fed female rats is in agreement with that seen by Cani et al. [44] wherein consumption of the prebiotic fiber, oligofructose, increased amylin in *ob/ob *mice. The change in these two anorexigenic hormones lends support for the lower energy intake observed in the HF rats both in periods of direct exposure to the prebiotic but also in the period where all rats consumed the HFHS diet. The increase in body weight and energy intake was greatest in HP rats when they were introduced to the HFHS diet suggesting that consuming the HP diet from weaning to early adulthood did not protect them against the obesigenic effects of the HFHS diet. In fact, although final body weight did not differ between C and HP rats, the markedly higher body fat in the HP rats is metabolically harmful and suggests that a significant shift in nutrient partitioning occurred in these rats. On the other hand by consuming the high prebiotic diet throughout growth, HF rats were protected against excessive weight gain and fat mass accumulation when challenged with the HFHS diet. And even though the HF rats entered the HFHS diet challenge at a lower body weight they did not catch up this weight during the high energy feeding as weight gain remained significantly lower in HF versus HP rats during the final six weeks of the study. The mechanisms responsible for these contrasting protective and detrimental effects are not completely know but could involve changes in metabolism.

The expression of certain genes involved in glucose and lipid metabolism was altered with diet and these could exert effects that ultimately influence body composition. HMG-CoA reductase is the rate-limiting enzyme in cholesterol synthesis. Serum cholesterol can be reduced by administration of HMG-CoA reductase inhibitors and these are often prescribed to achieve reductions in LDL cholesterol [45]. We observed a significant decrease in HMG-CoA reductase mRNA expression in the liver of rats fed a long-term diet of high fiber which persisted even after both groups consumed the HFHS diet. A HFHS diet has been shown to increase hepatic HMG-CoA reductase mRNA in rats [46]. Given that all of our rats were fed HFHS diet for the last 6 weeks of the study one might anticipate that HMG-CoA reductase would increase in all rats similarly but it is clear that the residual effects of the high fiber diet consumed throughout growth persisted in these animals to minimize this response.

Another potential drug target for lowering cholesterol is cholesterol 7α-hydroxylase [47]. Cholesterol can be incorporated into bile salts by cholesterol 7α-hydroxylase, the initial and rate-determining enzyme for bile synthesis [48]. We demonstrated that a high protein diet, when followed by a diet high in energy results in decreased expression of cholesterol 7α-hydroxylase mRNA which could be indicative of decreased incorporation of cholesterol into bile acids and the potential for longer-term impairment in cholesterol metabolism in these rats.

Glucokinase (GK) catalyzes the rate-limiting step in glycolysis, the phosphorylation of glucose to glucose-6-phospate. In the liver, GK activity determines the rate of glucose utilization and glycogen synthesis [49]. GK first appears in the liver of rats about 16 days after birth and reaches adult activity levels 10-12 days later [50]. There is little research on the effect of long-term high protein diets on glucokinase or GLUT2 activity. We demonstrated an increase in the expression of both genes in the liver due to a long-term weaning diet high in protein subsequently followed by the high energy diet. Although not demonstrated consistently, a high fat diet has been shown to increase hepatic GK mRNA [49,51]. While some would suggest that increased GK activity could improve hepatic insulin resistance [52], others have shown that long-term increased GK activity leads to glucose intolerance and hepatic lipid accumulation in mice [53]. Mice over-expressing GK were also more sensitive to the diabetogenic effects of a high fat diet, which supports the elevated triglyceride content seen in our HP rats. Whether or not the increased GK mRNA seen in our high protein fed rats also played a role in the overall exacerbated negative response they had to the HFHS diet warrants further investigation.

The fructose transporter, GLUT5, is expressed in greatest concentration in the small intestine, but is also expressed in the kidneys, skeletal muscle and certain areas of the brain [54]. In rats, GLUT5 expression remains low until the completion of weaning and a switch to solid food [54]. It has been shown in individuals with type 2 diabetes that GLUT5 expression is higher in skeletal muscle [55] and in the intestines [56]. However, these findings are not consistent and remain controversial [54]. GLUT5 synthesis is regulated very quickly by diet as consumption of fructose results in an increase in the transporter within a few hours [57]. In the intestine of our rats fed a high protein diet we see a general down-regulation in the hexose transporters which likely reflects the lower carbohydrate content of this diet.

When we previously examined the expression of genes controlling glucose and lipid metabolism in young rats (days 7, 14, 21, 28, 35 of age) weaned onto high protein or fiber diets, we observed minimal change in gene expression in the intestine, and in the liver a decrease solely in FAS mRNA with HP and HF compared to control [23]. On the other hand, the greater GLP-1 levels we observed in these same young rats with high fiber diet [23] did persist into adulthood as demonstrated in the current study. Considered together, these studies comparing early and late changes to the diets would suggest that while some metabolic pathways (satiety hormones) are influenced very early on by diet, others (expression of genes regulating lipid metabolism) are only exasperated by a high energy diet in adulthood. The greater hepatic triglyceride content and fat mass in the high protein rats in this study provide phenotypic evidence for changes occurring at the level of gene expression.

## Conclusions

Our research demonstrating a significant susceptibility to an obese phenotype in rats weaned onto a HP diet and then challenged in adulthood with a HFHS diet suggests that lasting changes result from altering the composition of the first solid food that is consumed throughout growth into early adulthood. While all rats in this study consumed the same high energy diet during the last 6 weeks of the intervention, distinct metabolic profiles remained evident from exposure to the different diets during growth. This would suggest that these changes, either long-lasting or perhaps permanent, ultimately influenced the adiposity response of these rats to a high energy challenge in adulthood. Overall, it appears that a long-term diet high in protein, when mismatched with a high energy challenge, has negative effects on body mass and hormones and genes involved in glucose and lipid metabolism. However, a fiber-enriched diet may provide some protection. Given the continued escalation in the rates of obesity worldwide, further research examining dietary composition during growth and the long-term protective or detrimental influences they exert are clearly warranted.

## Competing interests

The authors declare that they have no competing interests.

## Authors' contributions

ADM participated in the design of the study, carried out the plasma analysis and DEXA, performed statistical analysis and drafted the manuscript. LKE carried out the oral glucose tolerance test and interpreted the data. MCH participated in the oral glucose tolerance test and performed gene expression analysis. KT carried out the triglyceride and cholesterol analysis and the associated statistical analysis and interpretation of the data. RAR conceived of the study and participated in its design and coordination and edited the manuscript. All authors read and approved the final manuscript.
